# Clinical and imaging features of lymphomatosis cerebri: analysis of 8 cases and systematic review of the literature

**DOI:** 10.1007/s10238-023-01224-9

**Published:** 2023-11-18

**Authors:** Mengke Fan, Lu Zhao, Qingjiang Chen, Mingzhi Zhang, Xudong Zhang, Zhihao Yang, Suxiao Li, Yunfei Song

**Affiliations:** 1 Department of Oncology, The First Affiliated Hospital of Zhengzhou University, Νo. 1 Jianshe East Road, Zhengzhou, 450052 Henan China; 2 Lymphoma Diagnosis and Treatment Center of Henan Province, Zhengzhou, Henan China; 3 Department of Neurology, The First Affiliated Hospital of Zhengzhou University, Zhengzhou, Henan China; 4 Department of Imaging, The First Affiliated Hospital of Zhengzhou University, Zhengzhou, Henan China

**Keywords:** Lymphomatosis cerebri, Primary central nervous system lymphoma, Diffuse infiltrative lesion

## Abstract

Lymphomatosis cerebri (LC) is a rare type of primary central nervous system lymphoma with diffuse, nonenhancing infiltrative lesions and is often misdiagnosed. Our study aimed to investigate the clinical characteristics and prognosis of LC through analyzing patients from the literature and our own center, so as to improve early diagnosis and treatment. PubMed, Web of Science and our hospital databases were reviewed, and information on demographic, clinical, pathological, cerebrospinal fluid (CSF), neuroimaging and treatment options was extracted. Univariate survival analysis was conducted by generating survival curves and comparing them using the log-rank test. Multivariate analysis was performed using the Cox proportional hazards regression model to identify the prognostic predictors. A total of 81 patients (median age: 58 years; interquartile range, IQR: 50–66.5 years), 45 males and 36 females, were included. The most common symptoms were cognitive impairment (65.4%) and gait impairment (50.6%). Imaging studies indicated that all 81 patients had supratentorial structure involvement, and 93.8% (76/81) had bilateral hemisphere involvement. There were 53.3% (32/60) patients with CSF pleocytosis and 65% (39/60) patients with increased CSF protein levels. The median time of diagnosis was 4.8 months (IQR: 2.3–6.9 months). Compared with 4 (95% CI: 1.78–6.22) months for all 81 patients, the median OS was 20 (95% CI: 8.24–31.76) months for those who had chemotherapy plus radiotherapy. Multivariate Cox analysis revealed that chemoradiotherapy (HR: 0.12; 95% CI: 0.02–0.68) and higher CSF glucose level (HR: 0.01; 95% CI: 0.00–0.26) were inversely associated with death. The diagnosis of LC should be alerted when neuroimaging with bilateral hemispheric involvement and CSF abnormality with pleocytosis and increased protein. Once the diagnosis is confirmed, the combination of chemotherapy and radiotherapy can be considered if the patient’s physical condition permits.Journal standard instruction requires an unstructured abstract. Kindly check and confirm.We have checked and confirmed that there is no problem.

**Supplementary Information:**

The online version contains supplementary material available at 10.1007/s10238-023-01224-9.

## Introduction

Lymphomatosis cerebri (LC) is a rare subtype of primary central nervous system lymphoma (PCNSL) [1]. PCNSL is an unusual extranodal non-Hodgkin’s lymphoma [2], and brain magnetic resonance imaging (MRI) usually shows single or multiple mass-like lesions with varying degrees of mass effect and strong contrast enhancement [3]. The imaging features of LC are diffuse and nonenhancing brain parenchymal infiltration [4]. LC is easily misdiagnosed as other types of white matter lesions, and its clinical diagnosis is difficult, which easily causes treatment delay. This study aimed to systematically review 8 cases of LC diagnosed in our hospital and 73 cases reported in previous studies and to analyze the clinical features, imaging, pathology, outcome characteristics and prognostic factors of LC, thereby providing a reference for the early diagnosis and treatment of LC.

## Material and methods

*Search strategy and selection criteria* The references in this paper were retrieved from the PubMed database and Web of Science database from January 1990 to June 2022. Meaningful combinations were selected from the expressions "primary," "lymphomatosis cerebri," "central nervous system," "diffuse" and "nonenhanced" to be searched. Based on the information in the abstracts of the retrieved articles, they were filtered by the following criteria. This study was approved by the ethics committee of the First Affiliated Hospital of Zhengzhou University (2022-KY-0869–001).

*Inclusion criteria were as follows* (i) diffuse white matter lesions on brain MRI without significant enhancement on enhanced scan (patchy contrast enhancement is also allowed), and (ii) histopathological diagnosis of central nervous system lymphoma. Exclusion criteria were as follows: (i) obvious mass shadow on the first MRI and nodular enhancement on enhanced scan, (ii) patients with systemic lymphoma revealed by bone marrow biopsy or imaging or (iii) intravascular lymphoma. A total of 215 relevant articles were retrieved, and 61 articles meeting the criteria were screened out, among which 7 articles were excluded: 2 due to unavailability of articles and 5 due to insufficient information. A total of 54 articles were reviewed, and 73 patients met all inclusion criteria [1, 4–56].

**Fig. 1:**
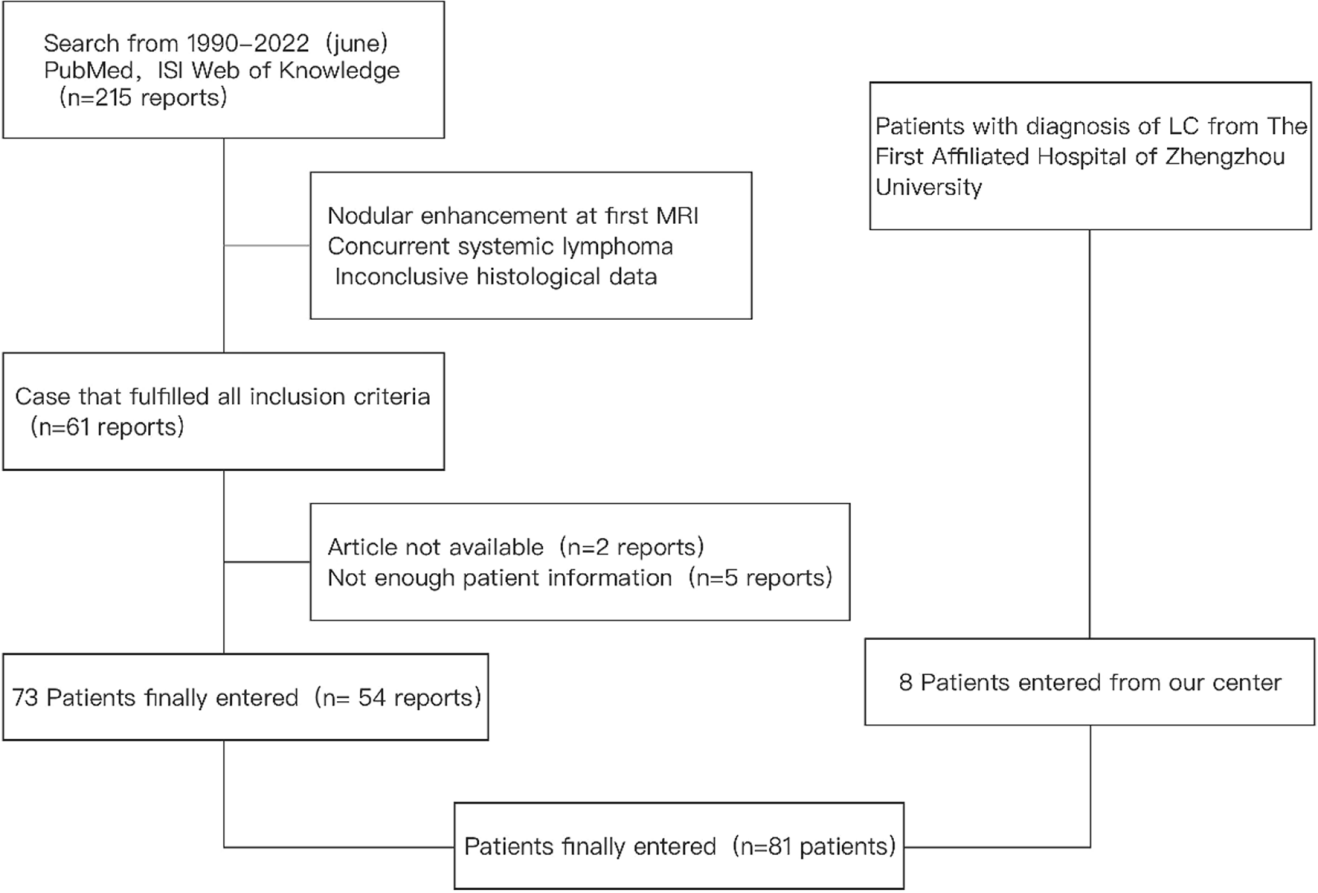
Flowchart showing search strategy performed

Ultimately, 73 cases from previous literature were included. In addition, 8 patients who met the above LC diagnostic criteria were screened out by screening the database of our center (Fig. 1).

*Data collection* The following data were collected: (i) epidemiology: age, sex, immunosuppression, (ii) imaging: lesion locations, T1 sequence, T2 sequence, magnetic resonance spectrum (MRS), diffusion-weighted imaging (DWI), enhancement, (iii) CSF: cell count, protein count and glucose level, (iv) clinical manifestations: karnofsky performance status (KPS) score at initial diagnosis (patients with KPS ≥ 70 were considered to be independent, and patients with KPS < 70 were considered to be dependent) and 9 categories of symptoms: behavior disturbance; consciousness disturbance; gait disturbance; sensory, motor, or cognitive impairment; language disorders; seizures; and headache, (v) treatment variables, which were divided into 4 categories: no treatment or corticosteroids only, chemotherapy alone, radiotherapy alone and combined chemoradiotherapy, (vi) pathological types and (vii) overall survival (OS), which was defined as the time from pathological diagnosis to the date of last follow-up or death, and the time to diagnosis was defined as the time from the first onset of symptoms to pathological diagnosis of LC.

*Statistical analysis* For descriptive data analysis, categorical variables were expressed as observed counts and percentages, and continuous variables were expressed as the mean or median (measurement data in line with a normal distribution were expressed as $\upchi$  ± s, and measurement data not in line with a normal distribution were described as median and interquartile).

Univariate survival analysis was made by survival curves and the log-rank test. Survival curves were generated using the Kaplan–Meier estimator and the log-rank test was used to compare the difference. Evaluated variables included general information (age, sex), clinical characteristics (KPS score, and time to diagnosis), imaging features, enhanced mode (lesion), histologic type (B cell lymphoma or T cell lymphoma), CSF parameters (glucose, protein and cell count) and treatment options. Multivariate analysis was performed using the Cox proportional hazards regression model to identify the predictors for survival. The cutoff level chosen for continuous variables was their median value. A *p* value less than 0.05 was considered significant. For all calculations, the software program SPSS 25.0 was used.

## Results

### Demographic characteristics

Eighty-one patients were included: 8 from our center and 73 from previous literature screening and analysis. The median age was 58 years (range: 28–80 years), and there was no significant difference in the sex ratio between patients: 36 females and 45 males (female/male ratio: 1:1.25). Only 4 patients were immunosuppressed (patients 5, 31, 36 and 57). The age of onset was significantly younger in patients with immune deficiencies than in the rest of patients (median age: 47 years; range: 41–69 years).

### Clinical features

Cognitive disturbance and gait disturbance were the most common symptoms in 53 (65.4%) and 41 (50.6%) patients, respectively, followed by behavior disturbance in 32 (39.5%) patients. A total of 12 patients presented with disturbance of consciousness. Headache occurred in 11 patients. The frequency of seizures was low, with only 6 cases (7.4%) reported. Five patients (6.2%) had diplopia. Among 81 patients, 57 patients (70.0%) had KPS < 70 at diagnosis.

### Neuroimaging

**Fig. 2:**
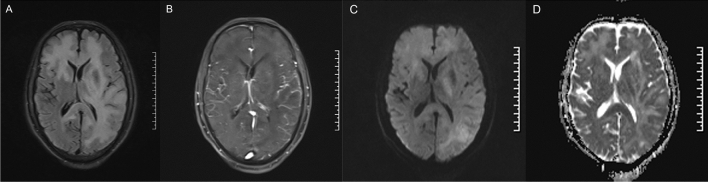
Brain MRI at initial diagnosis in a 75 year-old male patient. T2FLAIR, **A** showed hyperintensity in the right frontal lobe and the left frontal, parietal and temporal lobe, and T1 enhancement, **B** sequences showed patchy mild enhancement. DWI (**C**) and ADC (**D**) sequences showed hyperintensity lesions

All patients had supratentorial lesions, and 43 patients (53.1%) had both supratentorial and infratentorial infiltrates. Of note, only 5 patients (6.2%) had unilateral infiltration on the first MRI, and all the remaining patients had bilateral hemispheric involvement (Fig. 2). Brain MRI showed no enhancement pattern in 44 (54.3%) patients and punctate or patchy enhancement in 37 cases (45.7%), including 2 patients with specific enhancement patterns infiltrating along the corticospinal tract. Basal ganglia were involved in 47 of 67 patients (58.0%), and spinal cord infiltration was indicated in 7 of 30 patients by MRI or positron emission tomography computed tomography (PET-CT).

**Fig. 3:**
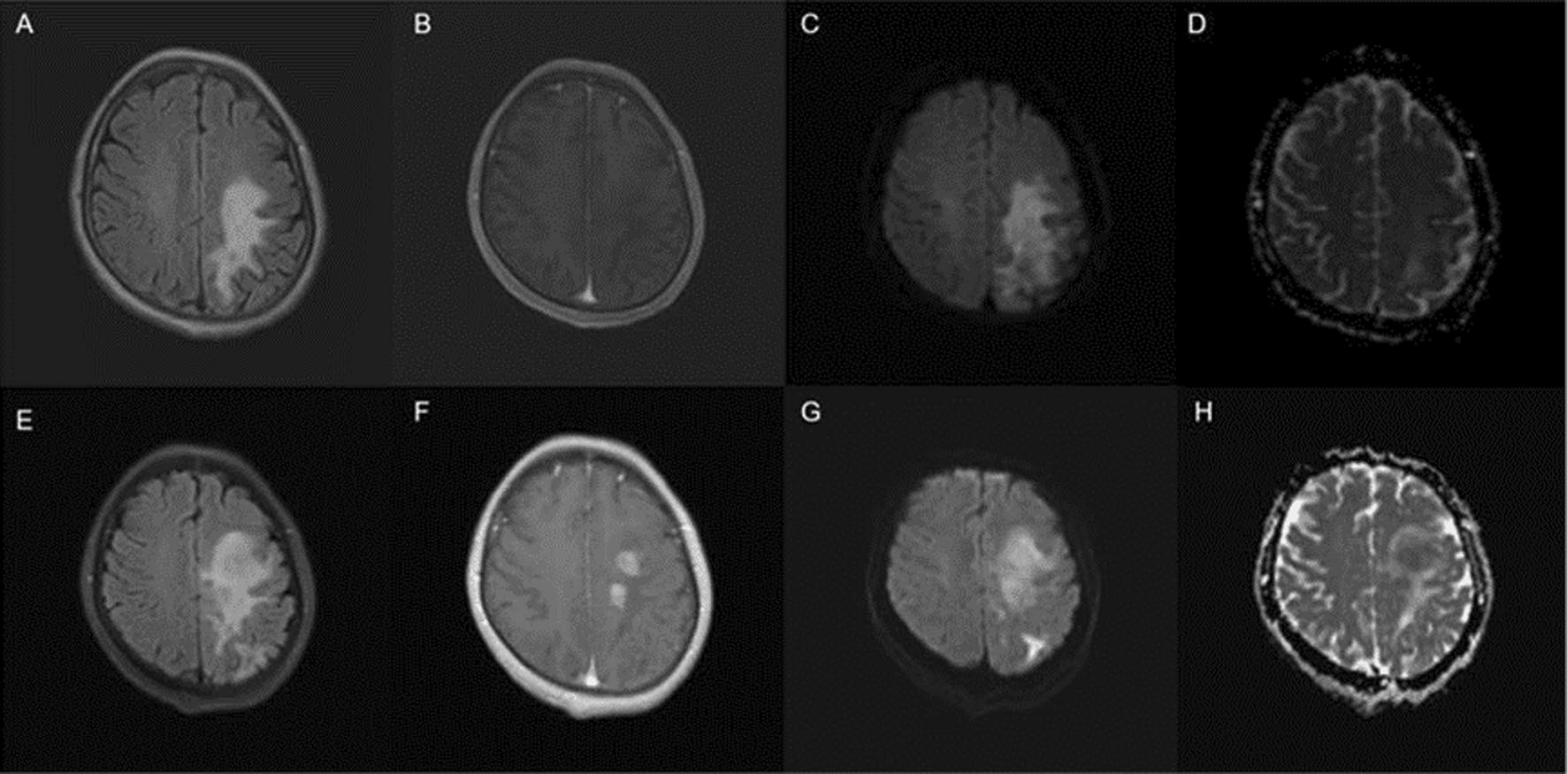
Brain MRI at initial diagnosis (**A**, **B**, **C** and **D**) and disease progression 6 months later (**E**, **F**, **G** and **H**) in a 56-year-old female patient. T2FLAIR (**A**) showed hyperintensity in the left frontoparietal lobe, with patchy mild enhancement on the T1 enhanced sequence (**B**). DWI (**C**) and ADC (**D**) sequences showed hyperintensity lesion. Brain MRI of 6 month follow-up showed a significantly enlarged range of abnormal signals on T2FLAIR (**E**) and obvious nodular enhancement on T1 enhanced sequences (**F**), hyperintense on DWI (**G**) and heterogeneous signal (hypointense of the center and hyperintense of the circum) on ADC sequences (**H**)

Twenty-five patients underwent follow-up MRI of the brain, and 19 patients showed some changes in contrast enhancement patterns, except for 4 patients who consistently showed no enhancement pattern and 2 patients who showed little change in patchy enhancement. Among them, 11 patients progressed from no initial enhancement to punctate or patchy enhancement, 6 patients had an increased patchy enhancement area (Fig. 3), and 2 patients progressed from patchy enhancement to mass-like enhancement (similar to the MRI findings of typical PCNSL).

MRS examination was performed in 9 patients, which showed an increase in Cho (*n* = 7), a decrease in NAA (*n* = 2), a decrease in Cr (*n* = 5) and an increase in lipids (*n* = 3). Twenty-five of the 36 patients (66.7%) showed high signal on diffusion-weighted imaging (DWI) brain MRI. Brain positron emission tomography fluorodeoxyglucose (PET-FDG) was performed in 26 patients, and only 10 showed increased deep white matter metabolism.

### Cerebrospinal fluid examination

CSF information was extracted from 60 patients. A total of 53.3% (32/60) of the patients had CSF pleocytosis; the protein level was increased in 39 cases (*n* = 60, 65.0%), and the glucose level was de creased in 8 cases (*n* = 54, 14.8%). CSF was normal in 10 patients (*n* = 54, 18.5%). Oligoclonal bands were detected in CSF in only one patient (*n* = 18, 5.6%). In 48 cases (*n* = 53, 90.6%), the CSF cytology was negative for malignant cells. Abnormal lymphocytes were detected by flow cytometry in only 2 cases (*n* = 10, 20%).

### Pathology

Brain biopsies were performed in 62 patients, and autopsies were performed in 19 patients. The median time from first symptom onset to pathological diagnosis (*n* = 62) was 4.8 months (range: 0.2–31 m). Most patients (*P* = 72/80; 90%) had diffuse large B cell lymphoma, 8 (10%) had T cell lymphoma, and in 1 case, details of the lymphoma subtype were missing. Of the 19 patients with diffuse large B cell lymphoma, only 2 (10.5%) had a germinal center origin, and the remaining 17 had a nongerminal center origin.

### Therapy

The vast majority of patients (*P* = 60/65, 92.3%) received some form of treatment. Twenty-one patients (32.3%) received corticosteroids alone, 23 patients (35.4%) had high-dose methotrexate or other drug chemotherapy, 8 patients (12.3%) had chemotherapy combined with radiotherapy, and 8 patients had radiotherapy alone (total dose range: 30–50 Gy). Five patients were untreated and 16 had no reported treatment.

### Survival

**Fig. 4:**
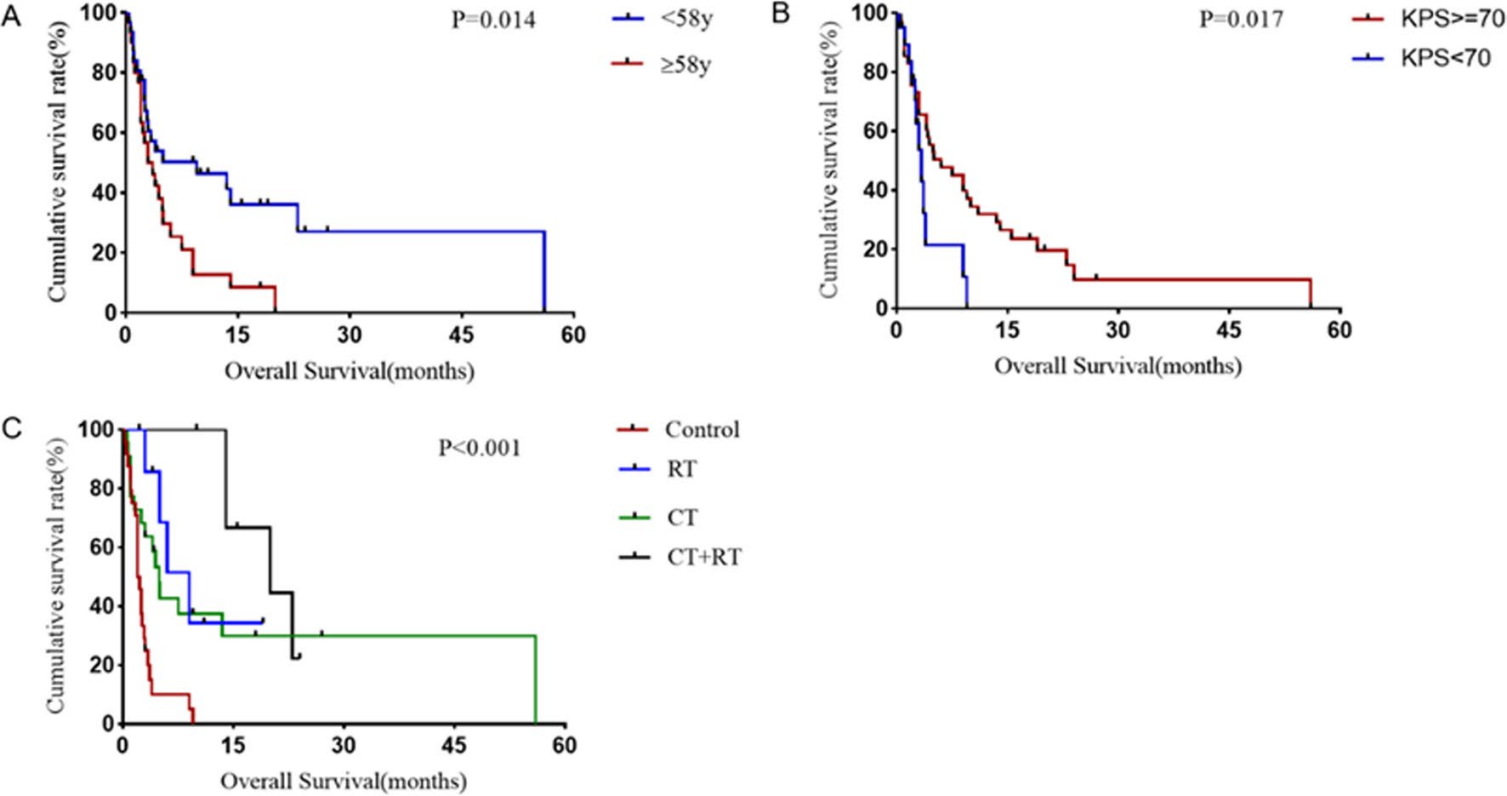
Kaplan‒Meier curves showing overall survival stratified by age, KPS score and treatment options in patients with lymphomatosis cerebri (no treatment or corticosteroids only = control, radiotherapy alone = RT, chemotherapy alone = CT and combined chemoradiotherapy = CT + RT)

The median OS was 4 (95% CI: 1.78–6.22) months. The majority (75.4%) of patients were dead at the time of reporting. The median OS was 2 (95% CI: 1.50–2.50) months for patients who had corticosteroids alone or not treated, and 4.9 (95% CI: 3.57–6.27) months for patients who had chemotherapy. Among patients who had radiotherapy alone, the median OS was 9 (95% CI: 4.54–13.46) months, compared with 20 (95% CI: 8.24–31.76) months for those who had chemotherapy plus radiotherapy. Only one patient underwent autologous stem cell transplantation as a consolidation therapy, with an OS of 13.5 months (Fig. 4).

Univariate survival analysis revealed that age ≤ 58 years (*P* = 0.014), KPS ≥ 70 at presentation (*P* = 0.017), CSF glucose level ≥ 0.51 g/L (*P* < 0.001) and combined chemoradiotherapy (*P* < 0.001) were associated with better OS (Table 1). Other clinical, pathological, radiological and CSF variables were not associated with survival outcomes.

**Table 1:** Clinical characteristics, examination and treatment effect on overall survival by subgroup

Factors	No	mOS(months)	*P* value
Gender			0.822
Male	34	3.4	
Female	27	4.9	
Age			0.014
≤ 58 years	31	9.5	
> 58 years	30	3.0	
Time to diagnosis			0.828
≤ 120 days	22	4.9	
> 120 days	19	13.5	
KPS			0.017
≥ 70	20	13.5	
< 70	41	3.0	
CSF cell count			0.648
> 5 cells	20	3.4	
≤ 5 cells	21	3.6	
CSF protein count			0.485
> 0.45 g/L	24	3.0	
≤ 0.45 g/L	16	9.0	
CSF glucose level			< 0.001
< 0.51 g/L	1	0.6	
≥ 0.51 g/L	33	4.0	
Pathology			0.245
B cell	57	3.9	
T cell	4	2.0	
Gd contrast enhancement			0.797
Yes	25	4.0	
No	36	3.4	
Treatment			< 0.001
Control	24	2.0	
CT	22	4.9	
RT	7	9.0	
CT + RT	8	20.0	

*KPS* Karnofsky performance status, *mOS* median overall survival, *CSF* cerebrospinal fluid, *RT* radiotherapy, *CT* chemotherapy, *ST* supratentorial, *IT* infratentorial, *Gd* gadolinium

**Fig. 5:**
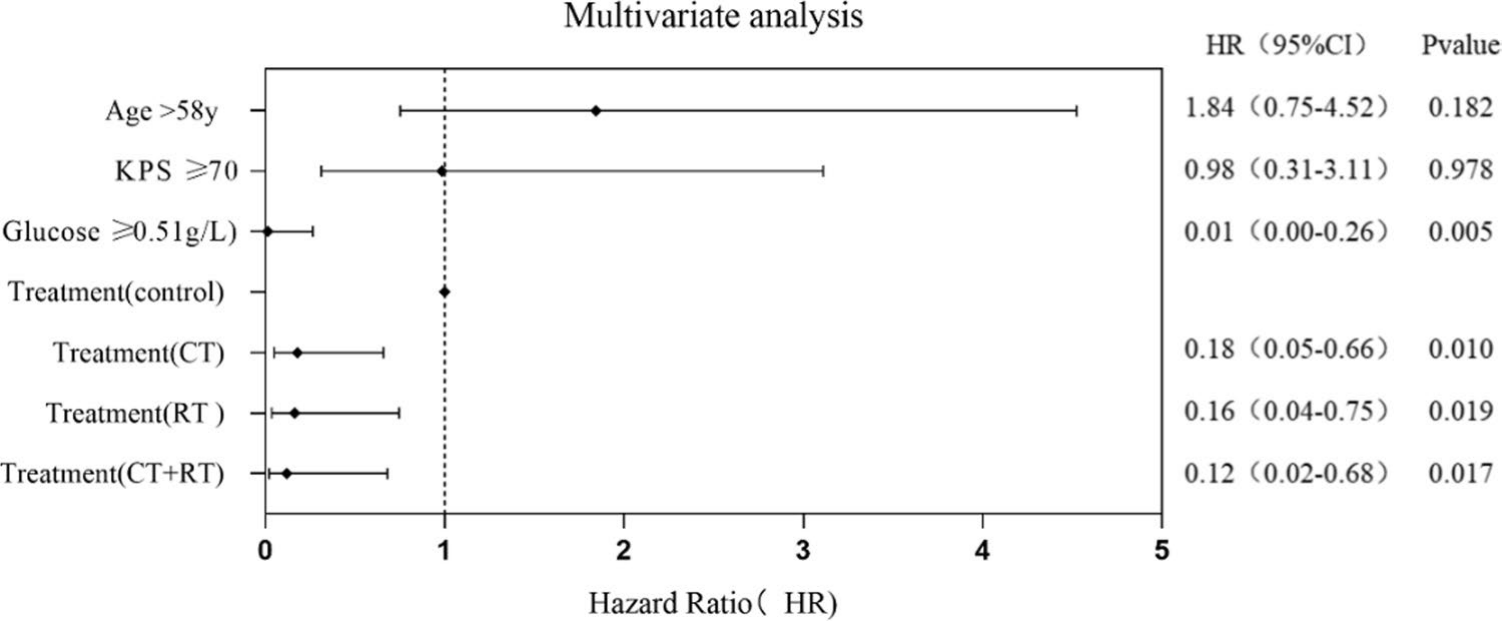
Multivariate Cox analysis of death in lymphomatosis cerebri patients (KPS: karnofsky performance status, control: no treatment or corticosteroids only, CT: radiotherapy, RT: chemotherapy)

The age of disease onset, KPS score at presentation, CSF glucose level and treatment options were included in multivariate Cox analysis, indicating that CSF glucose level ≥ 0.51 g/L (HR: 0.01; 95% CI: 0.00–0.26; *P* = 0.005), radiotherapy (HR: 0.16; 95% CI: 0.04–0.75; *P* = 0.019), chemotherapy (HR: 0.18; 95% CI: 0.05–0.66; *P* = 0.01) and radiotherapy plus chemotherapy (HR: 0.12; 95% CI: 0.02– 0.68; *P* = 0.017) were inversely associated with death, and KPS ≥ 70 (HR: 0.98; 95% CI: 0.31–3.11; *P* = 0.978) and age > 58 years (HR: 1.84; 95% CI: 0.75–4.52; *P* = 0.182) were not associated with death (Fig. 5).

## Discussion

LC is a rare form of brain lymphoma, and there are few studies on LC at present. So far, only a few studies have provided partial information for clinical characteristics, prognosis and treatment [4], and there has been almost no systematic review of LC in recent years, thus making clinical diagnosis and treatment extremely difficult. Our study conducted a systematic review and analysis of LC patients, which is the largest sample size study so far.

In our study, patients are more likely to be between 50 and 66 years old, and the proportion of men is slightly higher than that of women, which was not different from PCNSL. LC is a rare subtype of PCNSL. Compared with typical PCNSL (median diagnosis time: about 70 days) [4, 57], LC has a long delay from onset to definite pathological diagnosis (median diagnosis time: 143 days) due to its atypical imaging features and nonspecific clinical manifestations.

Most of the cases in this study had no or only small patchy enhancement at initial diagnosis and then patchy enhancement or increased patchy enhancement area at follow-up. This phenomenon may be related to the progressive pathological features of the diffuse infiltration of lymphoma cells into perivascular brain tissue [3]. It is generally believed that the pattern and mechanism of brain MRI contrast enhancement mainly depend on the permeability of the blood‒brain barrier (BBB). In LC patients, diffuse infiltration of tumor cells without significant BBB destruction showed a nonenhancement pattern or small patchy enhancement on imaging. As the disease progresses, the BBB becomes more impaired, which leads to an increase in the lesion enhancement area. Because of the nonspecificity of LC in imaging and clinical manifestations, LC often needs to be differentiated from gliomatosis cerebri (GC), progressive multifocal leukoencephalopathy (PML), infectious process, small vessel vasculopathy, toxic-metabolic disorder and other diseases. About half of patients with GC present with seizure [58, 59]. The patient characteristics of PML are that the CSF John Cunningham (JC) virus deoxyribonucleic acid (DNA) test is positive [60], small vascular disease shows punctate hyperintense on the T2 FLAIR sequence of MRI [61], and infection and toxic-metabolic disorder can be identified by relevant CSF or hematological markers. According to our study, clinical features combined with brain MRI examination may provide the basis for the diagnosis of LC. Cognitive decline and gait disturbances were seen in more than half of LC patients, and bilateral hemispheric diffuse involvement was the most prominent imaging feature of LC, which is not common in other solid brain tumors.

In CSF analysis, 53.3% of patients showed increased CSF cell levels, and 65% of patients showed increased protein levels, which may provide a reference for the diagnosis of LC. Ninety percent of patients presented with negative malignant cytology, suggesting that LC may rarely involve the leptomeninges. Therefore, clinicians should consider a definitive biopsy diagnosis rather than multiple lumbar punctures if CSF malignant cytology and flow cytometry are negative. In our study, low glucose level in CSF is considered to be a poor prognostic factor for survival, and the increased protein and cell count in CSF examination may indicate the diagnosis of LC. Due to the limited number of patients reported thus far, these indicators can only play a suggestive role in the diagnosis of LC and cannot be completely used as a diagnostic basis.

The prognosis of LC patients is extremely poor, the median overall survival (mOS) in this study was only 4 (95% CI: 1.78–6.22) months. In the process of a previous literature review, it was found that some scholars believe LC is the sentinel lesion of PCNSL. Our study argues that the available evidence does not support this point. First, in the review of this paper, among 25 patients who underwent follow-up imaging examination, only 2 cases progressed to classical mass-like enhancement. Moreover, the survival prognosis of LC was significantly worse than that of typical PCNSL [62]. In this study, the treatment options were identified as prognostic predictors. The mOS with several treatment regimens was observed and was found to be higher in the chemotherapy plus radiotherapy group (mOS = 20 months 95% CI: 8.24–31.76) than in the chemotherapy-alone group (mOS = 4.9 months 95% CI: 3.57–6.27) and radiotherapy-alone group (mOS = 9 months 95% CI: 4.54–13.46).

## Conclusion

In summary, our study outlines the demographic, clinical and radiological features of LC, which can provide reference for the diagnosis of LC. It should be noted that the differential diagnosis of LC should be considered when diffuse bilateral hemispheric involvement and elevated CSF cell and protein count are present in patients with no evidence of systemic or infectious disease. If enhanced MRI progression is observed during the observation period or the patient presents with progressive cognitive impairment or gait disturbance, timely biopsy should be performed to avoid a delay in diagnosis. In addition, for LC patients with tolerable physical conditions, chemotherapy combined with radiotherapy should be considered, which may effectively prolong the survival time.

### Supplementary Information

Below is the link to the electronic supplementary material.

Supplementary file1 (DOCX 42 KB)
