# Supplementary material for: Clinical and imaging features of lymphomatosis cerebri: analysis of 8 cases and systematic review of the literature
Source: Clin Exp Med. 2023 Nov 18;23(8):4673–80. doi: 10.1007/s10238-023-01224-9 (PMC10725345; doi:10.1007/s10238-023-01224-9)
Supplement: Supplementary file 1 — Supplementary file1 (DOCX 42 KB) [file 10238_2023_1224_MOESM1_ESM.docx]

| Patient | reference | Gender | Age（y） | KPS | Clinical presentation | Gd contrast enhancement | Localization | Time to diagnosis(days) | Treatment | Histology | Origin | Overall Survival(months) | Spinal cord involvement |
| --- | --- | --- | --- | --- | --- | --- | --- | --- | --- | --- | --- | --- | --- |
| 1 | 【11】 | F | 50 | <70 | M, W | YES | ST+IT |  | CT | B |  |  | YES |
| 2 | 【12】 | M | 64 | ≥70 | CS, CG, W, B, L | NO | ST+IT | 90 | CS | B |  | 3 | NO |
| 3 | 【9】 | F | 59 | <70 | CG, L, M, S, W | NO | ST+IT |  | CS, CT | B | Non-GCB | 4.43 |  |
| 4 | 【9】 | M | 43 | <70 | CG, W | NO | ST | 60 | CT | B | Non-GCB | 56 |  |
| 5 | 【9】 | M | 41 | <70 | H | NO | ST |  | None | B | Non-GCB | 2.62 |  |
| 6 | 【9】 | M | 61 | <70 | CG, W | NO | ST+IT | 30 | CT | B | Non-GCB | 4.92 |  |
| 7 | 【9】 | M | 31 | ≥70 | CG, W, SEI | YES | ST+IT | 138 | CS, CT, HDC/ASCT | B |  | 13.51 |  |
| 8 | 【9】 | M | 52 | ≥70 | B, CG, H | NO | ST | 39 | None | T |  | 1.08 |  |
| 9 | 【9】 | M | 69 | ≥70 | B, CG | YES | ST+IT |  | CS | B | Non-GCB | 3.93 |  |
| 10 | 【10】 | M | 45 | <70 | CG, W, B | NO | ST+IT |  |  | T |  |  | YES |
| 11 | 【10】 | M | 56 | ≥70 | CG, | NO | ST |  |  | B |  |  |  |
| 12 | 【10】 | M | 59 | <70 | H, W | YES | ST+IT |  |  | B |  |  |  |
| 13 | 【10】 | M | 78 | <70 | CG, W, B | YES | ST+IT |  |  | T |  |  | NO |
| 14 | 【10】 | F | 28 | <70 | CG, W, B | NO | ST |  |  | B |  |  | NO |
| 15 | 【10】 | M | 54 | ≥70 | - | YES | ST |  |  | B |  |  | NO |
| 16 | 【10】 | F | 58 | <70 | CG, W, B | NO | ST |  |  | B |  |  |  |
| 17 | 【10】 | M | 49 | <70 | CG, W, B | YES | ST+IT |  |  | B |  |  |  |
| 18 | 【10】 | M | 37 | <70 | CG, W | YES | ST+IT |  |  | B |  |  | NO |
| 19 | 【10】 | F | 59 | <70 | H, CG, W | NO | ST |  |  | B |  |  | NO |
| 20 | 【10】 | F | 62 | <70 | H, W | NO | ST+IT |  |  | T |  |  |  |
| 21 | 【13】 | F | 66 | <70 | H, CG, W | NO | ST | 270 | CS | B |  | 9 | NO |
| 22 | 【14】 | M | 46 | <70 | CG, W, L | NO | ST+IT | 285 | CS, CT | B |  | 9.5 | NO |
| 23 | 【15】 | M | 67 | ≥70 | CS, CG | YES | ST | 120 | CS, CT | B |  | 3 | NO |
| 24 | 【16】 | F | 67 | <70 | L, W | YES | ST+IT |  |  | B |  |  |  |
| 25 | 【16】 | M | 71 | <70 | W, M, CG | YES | ST+IT |  |  | B |  |  |  |
| 26 | 【17】 | F | 56 | <70 | CS, L | YES | ST+IT | 74 | CS+CT | B | Non-GCB | 4 | YES |
| 27 | 【18】 | M | 62 | <70 | CS, M, W | YES | ST+IT |  | CS | B |  | 1.67 |  |
| 28 | 【20】 | F | 60 | <70 | L, W, M | NO | ST+IT | 300 | CT | B | Non-GCB | 1 | NO |
| 29 | 【21】 | M | 72 | ≥70 | W, M, CG | NO | ST+IT | 111 | CS | B | Non-GCB |  |  |
| 30 | 【22】 | F | 51 | <70 | B, W, CG | YES | ST | 44 | CT | B |  | 18 | YES |
| 31 | 【23】 | F | 48 | <70 | M, H, SEI | YES | ST |  | CS | B |  | 2.87 | NO |
| 32 | 【24】 | F | 58 | <70 | M | NO | ST+IT |  | CS | B |  | 3.39 | NO |
| 33 | 【25】 | F | 66 | <70 | M | NO | ST | 90 | CS+RT | B | Non-GCB | 9 | NO |
| 34 | 【26】 | M | 53 | ≥70 | B, CG | NO | ST+IT | 120 | CS+RT | B | Non-GCB | 11 | NO |
| 35 | 【27】 | F | 68 | ≥70 | B, CG | YES | ST+IT | 5 | CT+RT | B |  | 14 |  |
| 36 | 【28】 | F | 73 | <70 | B, CG, H, M | NO | ST+IT | 150 | CS+RT | B |  | 4 |  |
| 37 | 【29】 | F | 78 | <70 | CS | YES | ST |  | CS | B |  | 3 |  |
| 38 | 【1】 | F | 50 | ≥70 | B, CG, L | NO | ST+IT | 201 | RT+CT | B |  | 10 | NO |
| 39 | 【30】 | M | 37 | ≥70 | M, S | NO | ST+IT | 240 | CS+CT+RT | B |  | 24 | YES |
| 40 | 【31】 | M | 69 | <70 | CG | YES | ST+IT |  | NONE | B |  | 2 | YES |
| 41 | 【32】 | F | 56 | <70 | B, CG | YES | ST+IT | 150 | CS+RT | B |  | 19 |  |
| 42 | 【33】 | M | 74 | <70 | B, CG | YES | ST | 360 | CT | B |  | 18 | NO |
| 43 | 【34】 | M | 67 | <70 | B, CG, M, L, W | YES | ST+IT |  | CS | B | GCB | 0.7 | NO |
| 44 | 【35】 | F | 56 | <70 | CG, B, M, W, L | YES | ST+IT | 285 | CS+CT | B |  | 0.7 |  |
| 45 | 【36】 | F | 65 | <70 | B, M, CG | NO | ST | 120 | CS+CT | B |  | 3 |  |
| 46 | 【37】 | M | 69 | ≥70 | L | NO | ST+IT |  | CS | B |  | 2.27 |  |
| 47 | 【38】 | M | 65 | <70 | CG, L, M, W | YES | ST+IT | 195 | CS+CT | B |  | 7.5 |  |
| 48 | 【38】 | F | 80 | <70 | CS, B, CG, L, M | NO | ST+IT |  | CS | B |  | 1.17 |  |
| 49 | 【38】 | M | 62 | <70 | B, M, W, L | YES | ST | 60 | CS | B |  | 0.33 |  |
| 50 | 【39】 | M | 57 | ≥70 | CG, W | NO | ST+IT |  | CS | B |  | 2 | NO |
| 51 | 【40】 | M | 49 | <70 | B, H, L, W, CG | NO | ST | 240 | CS | B |  | 0.49 |  |
| 52 | 【41】 | M | 41 | ≥70 | B, CG | NO | ST+IT |  | CS | B |  | 2.5 | NO |
| 53 | 【41】 | F | 75 | <70 | B, CG | NO | ST | 150 |  | B |  |  |  |
| 54 | 【42】 | M | 55 | <70 | B, CG, W | NO | ST+IT | 90 | CS+CT+RT | B |  | 2.23 |  |
| 55 | 【43】 | M | 75 | ≥70 | CG | YES | ST | 74 | CS+RT | T |  | 6 |  |
| 56 | 【44】 | F | 57 | ≥70 | B, CG, L, M, W | YES | ST | 150 |  | T |  |  | NO |
| 57 | 【45】 | M | 71 | <70 | CS, CG, W | YES | ST | 68 | CS | B |  | 3.6 | NO |
| 58 | 【46】 | M | 61 | <70 | CS, CG, B, L | NO | ST | 165 | CS | B |  | 2.5 | NO |
| 59 | 【47】 | M | 59 | <70 | CG | NO | ST+IT |  | CS | B |  | 2 | NO |
| 60 | 【48】 | F | 64 | <70 | CG, W | NO | ST+IT |  | NONE | B |  | 2 |  |
| 61 | 【49】 | F | 60 | ≥70 | CS, B, CG, L, M | NO | ST |  | NONE | T |  | 2 |  |
| 62 | 【50】 | M | 54 | ≥70 | M, L, B | YES | ST | 210 | CS+RT | T |  | 5 |  |
| 63 | 【51】 | F | 76 | <70 | B, CG, L | NO | ST | UK | CS | B |  | 1 |  |
| 64 | 【52】 | M | 39 | <70 | M, L, W, SEI | NO | ST+IT | 90 | CS+RT | B |  | 3 |  |
| 65 | 【53】 | F | 35 | ≥70 | H, S | NO | ST | 930 | CS+CT+RT | B |  | 14 | YES |
| 66 | 【54】 | F | 58 | <70 | M, L, S, W | NO | ST+IT | 210 | CS+CT | B |  | 1 |  |
| 67 | 【55】 | F | 45 | ≥70 | M, L, | NO | ST | 150 | CS+CT | B |  | 9 | NO |
| 68 | 【60】 | F | 66 | <70 | CG, W, | YES | ST+IT | 810 |  | B |  |  |  |
| 69 | 【61】 | M | 35 | <70 | SEI, CG, | NO | ST | 365 | CS, RT | B |  |  |  |
| 70 | 【58】 | M | 52 | <70 | L, W, M, S | NO | ST |  | CS | B |  |  | NO |
| 71 | 【59】 | F | 74 | <70 | H, L, CG, B | NO | ST | 43 | CS+RT | UK |  | 0.57 | NO |
| 72 | 【56】 | M | 48 | ≥70 | CS, CG | NO | ST+IT | 180 | CS+CT+RT | B |  | 23 |  |
| 73 | 【57】 | M | 61 | <70 | SEI, CG, | NO | ST | UK | CS+CT+RT | B |  | 20 | NO |
| 74 |  | M | 75 | <70 | L | NO | ST | 167 | CT+CS | B | Non-GCB | 5 | NO |
| 75 |  | F | 58 | ≥70 | CS, W, | YES | ST | 51 | CT+CS | B | Non-GCB | 27 | NO |
| 76 |  | F | 53 | <70 | W, SEI | YES | ST | 28 | CT+CS | B | Non-GCB | 1 | NO |
| 77 |  | F | 52 | <70 | B, CG | YES | ST+IT | 148 | CT+CS | B | Non-GCB | 1.5 | NO |
| 78 |  | M | 52 | <70 | B, L, W | YES | ST+IT | 50 | CT+CS | B | Non-GCB | 4.2 | NO |
| 79 |  | F | 56 | <70 | L, W | NO | ST | 203 | CT+CS | B | Non-GCB | 9.5 | NO |
| 80 |  | M | 29 | ≥70 | CS, CG | YES | ST | 37 | CT+CS+RT | B | Non-GCB | 15.5 | NO |
| 81 |  | M | 50 | <70 | L, M | YES | ST | 120 | CT | B | GCB | 2.5 | NO |

F: Female; M: Male; KPS: Karnofsky Performance Status; B: Behavioral; CG: Cognitive; H: Headache; L: Language; M: Motor; S: Sensory; W: Trouble walking; CS: consciousness; Y: Yes; N: No; Gd: Gadolinium; ST: Supratentorial; IT: Infratentorial; 1B: B-cell lymphoma; T: T-cell lymphoma; GCB: Germinal Center B-cell immunophenotype.
